# Association between social dominance hierarchy and PACAP expression in the extended amygdala, corticosterone, and behavior in C57BL/6 male mice

**DOI:** 10.1038/s41598-024-59459-9

**Published:** 2024-04-18

**Authors:** Edward G. Meloni, William A. Carlezon, Vadim Y. Bolshakov

**Affiliations:** 1 Department of Psychiatry, Harvard Medical School and McLean Hospital, Belmont, MA 02478 USA; 2 McLean Hospital, Mailman Research Center, 115 Mill St., Belmont, MA 02478 USA

**Keywords:** Social dominance, PACAP, BNST, Amygdala, Corticosterone, Startle, Social behaviour, Stress and resilience

## Abstract

The natural alignment of animals into social dominance hierarchies produces adaptive, and potentially maladaptive, changes in the brain that influence health and behavior. Aggressive and submissive behaviors assumed by animals through dominance interactions engage stress-dependent neural and hormonal systems that have been shown to correspond with social rank. Here, we examined the association between social dominance hierarchy status established within cages of group-housed mice and the expression of the stress peptide PACAP in the bed nucleus of the stria terminalis (BNST) and central nucleus of the amygdala (CeA). We also examined the relationship between social dominance rank and blood corticosterone (CORT) levels, body weight, motor coordination (rotorod) and acoustic startle. Male C57BL/6 mice were ranked as either Dominant, Submissive, or Intermediate based on counts of aggressive/submissive encounters assessed at 12 weeks-old following a change in homecage conditions. PACAP expression was significantly higher in the BNST, but not the CeA, of Submissive mice compared to the other groups. CORT levels were lowest in Submissive mice and appeared to reflect a blunted response following events where dominance status is recapitulated. Together, these data reveal changes in specific neural/neuroendocrine systems that are predominant in animals of lowest social dominance rank, and implicate PACAP in brain adaptations that occur through the development of social dominance hierarchies.

## Introduction

Stratification of social status has important implications for behavior and emotional health in both animals and humans, with individuals lower in social standing generally experiencing worse outcomes than those with higher standing^1–4^. Chronic psychosocial stress and agonistic interactions experienced by those in subordinate roles likely underlies this strong correlation between social rank and health^5,6^. In laboratory mice, several different paradigms have been used to examine differences in neural systems and substrates that may correlate with the assignment of animals as either dominant (e.g. alpha animals) or subordinate (e.g. beta, gamma, delta animals) in social dominance ranking constructs^7–9^. These include behavioral assays such as the tube test, territory urine marking, and the warm spot test where induced conflict and ensuing dominance among animals determines rank order^10–12^. One of the most commonly used assays to identify social dominance rank is through the observation and scoring of agonistic behavior of group-housed animals in a homecage setting^10,12–14^. This method relies on the natural, self-organizing dominance hierarchies that can develop in many strains of group-housed cages of mice as animals engage in periodic offensive (e.g. aggressive) and defensive (e.g. submissive) behaviors through the course of standard laboratory animal housing^14^. Agonistic behaviors can be elicited spontaneously, at the onset of the diurnal active period (e.g. lights-off), or after a simple perturbation such as a change into a new unfamiliar primary enclosure (e.g. cage change)^15^. Once established, hierarches are generally stable with especially high maintenance of rank over time for cagemates identified as either most- or least-dominant among group housed animals^7,16,17^. Using this methodology, several studies implicate medial prefrontal cortex- and nucleus accumbens-dependent neural circuits that influence social dominance rank^12,18–20^. Further, alterations in neuropeptides, hormones, genes and inflammatory biomarkers that are differentially expressed in dominant versus subordinate cagemates have been identified^14,15,21–24^. Of interest to our own work studying stress peptides such as corticotropin releasing factor (CRF)^25–27^, previous studies have shown alterations in CRF mRNA levels in dominant versus subordinate animals^14,24^, suggesting a relationship between social dominance and this important regulator of stress and social behavior^28–30^.

Along with CRF, another neuropeptide—pituitary adenylate cyclase-activating polypeptide (PACAP)—has been implicated in brain adaptations to stress^31–33^ and regulation of CRF and stress hormones such as corticosterone (CORT) through the hypothalamic–pituitary–adrenal (HPA) axis^34–36^. PACAP belongs to the secretin/glucagon superfamily of peptides and exists in two biologically active forms (as 38- and 27-amino acid peptides) found in peripheral tissues and brain^37^. PACAP-38 is the predominant form in the brain and shares identical amino acid sequence homology in species including mice, rats, sheep, and humans, indicating strong evolutionary conservation^38^. The high density of PACAPergic afferents and PACAP-type-I receptors (PAC1Rs) within the extended amygdala (e.g. central nucleus of the amygdala [CeA] and bed nucleus of the stria terminalis [BNST])^39–41^ suggests that this peptide plays a role in modulating neural activity related to psychological stress as well as experience-dependent learning^42–44^. We have demonstrated that PACAP influences AMPA receptor-dependent synaptic transmission in the CeA^45^, which receives direct PACAPergic innervation from the parabrachial nucleus^46^ and where PACAP may be endogenously released in response to pain. Further, we found that exogenously administered PACAP can affect expression of fear-related behavior and CORT levels in a fear-conditioning paradigm^47,48^, cause persistent alterations in sleep architecture^49^, and impact motivation and social behavior^50^. The current study was designed to examine differences in PACAP expression within the extended amygdala in cagemates of mice ranked according to social dominance interactions. We also examined the impact of social dominance rank on acoustic startle response, a behavioral test that is sensitive to stress and emotional state and is influenced by PACAP^51,52^, and tested the mice to ensure that differences in motor capabilities could not explain the formation of the hierarchies. Given that preclinical paradigms have been useful tools to help understand the neurobiology of emotional disorders that may arise from repeated psychological or physical stressors, understanding how PACAP systems influence social dominance behavior may have face and construct validity for studying these illnesses as they appear in humans^4,18,53^.

## Methods and materials

### Animals

**Figure 1:**
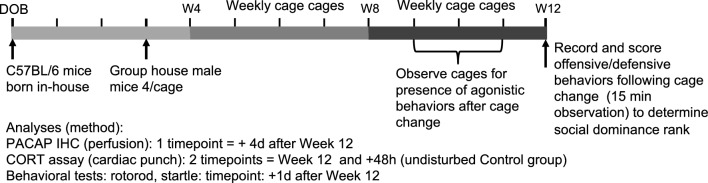
Timeline of the experimental design used in this study, including timepoints of various procedures used following the Week 12 (W12) observation of home-cage social dominance interactions; see Supplemental Fig. 1 for ethogram of offensive/defensive behaviors that were scored to determine rank. *PACAP* Pituitary adenylate cyclase-activating polypeptide, *IHC* immunohistochemistry, *CORT* corticosterone, *DOB* date of birth.

Male C57BL/6 mice bred and housed in the McLean Hospital vivarium were used. To generate the experimental animals, timed breeding pairs were established to allow pooling of male offspring into group cages of 4 mice/cage at approximately 3 weeks of age. At the time of group housing, the body weight of each mouse was determined and four animals of similar weight were housed together in each polycarbonate cage (28 × 18.5 × 12.5 cm) with laboratory bedding (Alpha Chip; Northeastern Products Co.) and one square (5 × 5 cm) of nesting material (Nestlet; Ancare). All mice were maintained on 12/12 h light dark cycles (lights on at 700 h) and food and water were provided ad libitum*.* Weekly cage changes to place animals into a clean homecage with new bedding and nesting material occurred on the same day each week always between 1000 and 1300 h. The timeline of all procedures is illustrated in Fig. 1. All animal procedures were approved by McLean Hospital’s Institutional Animal Care and Use Committee (Office of Laboratory Animal Welfare Assurance number A3685-01) in accordance with the National Institute of Health *Guide for the Care and Use of Laboratory Animals (8*^*th*^* Edition)*. The study was performed and results reported in accordance with ARRIVE guidelines.

### Social interaction measurement and determination of social dominance hierarchy.

Starting at 10 weeks of age, each the 4 group-housed mice were weighed and tails were marked with distinctive markings to individually identify each animal prior to cage change. Mice were observed for the presence of agonistic behaviors (see below) at this time point and cages where this behavior was not present were omitted from further study. At 12 weeks of age, immediately following the introduction of each group of mice into a new clean homecage, social interactions among the group-housed mice were video recorded for 15 min. A digital camera was placed above the center of the cage and used to record behavior for scoring of offensive (aggressive) and defensive (submissive) behavior using previously described methods to identify dominant and submissive mice^13,14,54^. A description of the types of agonistic behaviors used to rank animals according to an overall score in a matrix of offensive versus defensive behaviors is illustrated in Supplemental Fig. S1A. By assigning a “point” to each mouse in the cage for every offensive and defensive behavior it engaged in over the 15 min period, they could be ranked as Dominant or Submissive (e.g. having the highest score for offensive behaviors and lowest score for defensive behaviors or vice versa, respectively; see Supplemental Fig. S1B). As described in Horii et al.^14^, we identified a third rank of mice as “Intermediate” describing mice with offensive/defensive scores in-between those identified as dominant and submissive. However, unlike the study of Horii et al.^14^ which excluded this group from analyses, we retained these intermediates for our analyses as a third comparator group comprising the two animals in the cage of four that were ranked neither as Dominant nor Submissive. A separate cohort of 2 cages of mice (n = 4/cage) were solely used to track and demonstrate the stability of identified social dominance rank over time by recording agonistic behavior (15 min sessions) following cage change for an additional 3 weeks (Week 13–15; Supplemental Fig. S1C).

### PACAP immunohistochemistry (IHC)

Four days after the week 12 cage change and recording of social interaction to determine social dominance rank, cages of mice were overdosed with sodium pentobarbital (130 mg/kg; IP) and upon loss of toe-pinch reflex they were perfused intracardially with 0.9% saline (25 ml) followed by 2% paraformaldehyde, 0.05% gluteraldehyde, and 0.2% picric acid in 0.1 M PBS (75 ml) pH7.4. The execution of successful perfusion and fixation, which is critical for having confidence in the reproducibility of IHC assays, was evaluated by confirmation of three visual observations: tail curl within 1 min following introduction of the fixative solution, rigor in the head, legs and arms following perfusion, and the presence of yellow coloring (provided by the picric acid in the fixative) in the brain during removal. As a result, two mice in the Intermediate group were judged to have had poor perfusions and were removed from analysis. Brains were removed, stored for 3 d in a 30% sucrose/0.1 M PBS solution, and then cut serially in 30 µm coronal sections with every-other section from the BNST, and every-third section from the CeA placed in a 2-ml borosilicate glass vial (12 sections/vial for both brain areas) for processing of PACAP immunohistochemistry. All incubations were done on a rocker platform at room temperature. Sections were incubated in 0.6% hydrogen peroxide in 0.1 M PBS for 30 min followed by 3 washes (5 min) in 0.1 M PBS. Sections were preincubated in antibody medium (2% normal donkey serum, 1% bovine serum albumin, 00.3% Triton-X-100 in 0.1 M PBS) for 2 h followed by incubation for 24 h with a rabbit polyclonal antibody against PACAP (1:1000; Penninsula Labs; #T-4473) diluted in antibody medium. The sections were washed with PBS and incubated for 1 h with a donkey anti-rabbit biotinylated secondary antibody (1:400; minimum species cross-reactivity; Jackson ImmunoResearch). The sections were washed with PBS and incubated for 30 min with the avidin–biotin complex (Vector Laboratories, Burlingame CA) and then incubated for 5 min in 3,3’-diaminobenzidine (DAB)/H_2_O_2_ (Sigma Fast; Sigma) as a chromogen for visualization of PACAP peptide through the BNST and CeA. Processed sections were mounted on microscope slides and coverslipped with Permount (Fisher Scientific, Pittsburgh, PA) and observed with a Zeiss Axioscope 2 (Zeiss, Oberkochen, Germany). Still frame images of three coronal sections from each brain were captured approximating BNST and CeA regions from the mouse brain corresponding to images 52, 53, and 54 (BNSTov) and 69, 70 and 71 (CeAL) from the Allen mouse brain atlas (mouse.brain-map.org)^98,99^. As the CeAL has a much longer rostro-caudal extent than the BNSTov, we focused on caudal sections of the CeAL, which has denser PACAP expression than rostral sections (see Supplemental Fig. S2). PACAP immunoreactivity in each brain area was quantified by unbiased measurements of the optical density (O.D.) of pixels using ImageJ software for Macintosh (Scion Corp, Fredrick, MD, USA); ImageJ is a public domain, JAVA-based image processing program developed by the National Institutes of Health (NIH). Captured sections were switched to gray scale for threshold adjustments (scale 0–141 units normalized to background values of white matter) and mean values of O.D. were calculated from 0.1 mm^2^ regions with a fixed template placed within each of the brain areas (see Supplemental Fig. S2 for representative demarcation of areas measured). Some alternate sections through the BNST and CeA were also labelled for PACAP using immunofluorescence according to previously published methods^48^ for illustration purposes and to further delineate the boundaries of the BNSTov and CeAL (Supplemental Fig. S2) but were not used for quantification of PACAP in these regions.

### Corticosterone assay

Serum CORT levels were measured following cage change and the 15 min session for recording of agonistic behavior in a separate cohort of group-housed 12 week-old mice to determine the impact of agonistic behavior on this stress-dependent hormone in animals of different social rank. A separate cohort of animals was used as a control group for the stress of cage change and accompanying agonistic behaviors by collecting blood in undisturbed cages of mice 48 h after the 12 week cage change and recording of social interaction used to determine social dominance rank in this cohort. Given diurnal variations in mouse CORT levels, blood sampling was conducted between 1000 and 1100 h, when CORT levels are stably low^55,56^. Mice were overdosed with sodium pentobarbital (130 mg/kg; IP) and upon loss of toe-pinch reflex, the chest cavity was opened. A 0.5 cc Insulin syringe (U-100 syringe; Becton-Dickson, Franklin Lakes, NJ) with a 28 gauge needle (0.5 in length) was used to draw blood from the right ventricle of the heart. This procedure is rapid (< 3 min) and previous work indicates that it is unlikely that anesthesia significantly impacts CORT levels on this time scale^57^ although others have found that longer exposures to pentobarbital anesthesia can affect CORT responses in rats^58^. Blood was transferred to a sterile 2 ml serum blood collection tube (BD Vacutainer; Becton-Dickson, Franklin Lakes, NJ) and allowed to clot at room temperature for 30 min before centrifugation for 10 min at 3000 rpm. Serum was removed, aliquoted, and stored at − 80 °C until assayed by ELISA following the manufacturer’s directions for quantitative determination of CORT levels in rat/mouse serum (Alpco Diagnostics, Salem NH). All samples were loaded onto a single plate in duplicate wells and assayed using a BioTek Synergy HT microplate reader to compare samples against a standard curve of known mouse CORT concentrations. The sensitivity of the assay was 6.1 ng/ml.

### Rotorod

To explore whether motor capacities differ among groups—a factor that could potentially be involved in how the social hierarchies form—mice were tested on the accelerating rotorod (Ugo Basile; RotaRod model 7750) one day after the 12 week cage change and recording of social interaction among the group-housed mice. The four mice from each cage were placed on the rotorod cylinders at the same time starting at a slow rotational speed (4 rpm) which gradually increases over 2 min to a maximum of 40 rpm. Each lane on the device is equipped with individual timers to record latency-to-fall with a maximum trial length of 3 min. Mice are tested three times with a 5 min interval between tests with an overall latency-to-fall score averaged across the three trials for each mouse. Following this test, mice were perfused as described above for analysis of PACAP IHC.

### Acoustic startle

A separate cohort of mice was tested for acoustic startle one day after the 12 week cage change and recording of social interaction among the group-housed mice. Testing was conducted in 4 identical mouse startle cages consisting of 6 × 6 × 5 cm Plexiglas cages with metal rod flooring attached to a load-cell platform. Both the startle cages and platform were located within a 69 × 36 × 42 cm fan-ventilated sound-attenuating chamber (Med Associates, Georgia, VT). Cage movement resulted in a displacement of a transducer in the platform where the resultant voltage was amplified and digitized on a scale of 0 to ± 2000 arbitrary units by an analog-to-digital converter card interfaced to a personal computer (PC). Startle amplitude was proportional to the amount of cage movement and defined as the maximum peak-to-peak voltage that occurred during the first 200 ms after the onset of the startle stimulus. Constant wide-band background noise (60 dB; 10–20 kHz) and 50 ms startle stimuli (1–32 kHz white noise, 5 ms rise/decay) were generated by an audio stimulator (Med Associates) and delivered through speakers located 7 cm behind the startle cage. The calibration, presentation, and sequencing of all stimuli were under the control of the PC using specially designed software (Med Associates). For testing, mice were placed in the startle chambers and given a 5 min acclimation period followed by presentation of two habituating startle stimuli (100 dB, 30 s interstimulus interval; ISI). Mice were then presented with 30 startle stimuli at three different intensities (95, 100, 105 dB); the 10 trials at each intensity were presented in a semirandom order with a 30 s ISI. Startle amplitude data were expressed as the mean averaged across the 10 trials for each of the three startle-eliciting intensities.

### Statistical analyses

Data are presented as means ± standard error (SEM) for mice ranked as Dominant, Intermediate, or Submissive based on their agonistic behavior scores following the Week-12 cage change. The impact of social dominance rank on PACAP expression in the BNSTov and CeAL and on blood CORT levels was analyzed using a two-way ANOVA with rank (Dominant, Intermediate, Submissive) as a between-subjects factor. Additional comparisons of social rank on PACAP and CORT were further carried out as appropriate with one-way ANOVAs with subsequent post-hoc comparisons using Sheffe’s test. Body weight and rotorod performance was analyzed using separate independent-measures one-way ANOVAs. Startle data were analyzed using a two-way ANOVA with rank (Dominant, Intermediate, Submissive) as a between-subjects factor and startle intensity (95, 100, 105 dB) as a within-subjects factor.

## Results

### PACAP IHC, body weight and rotorod performance

**Figure 2:**
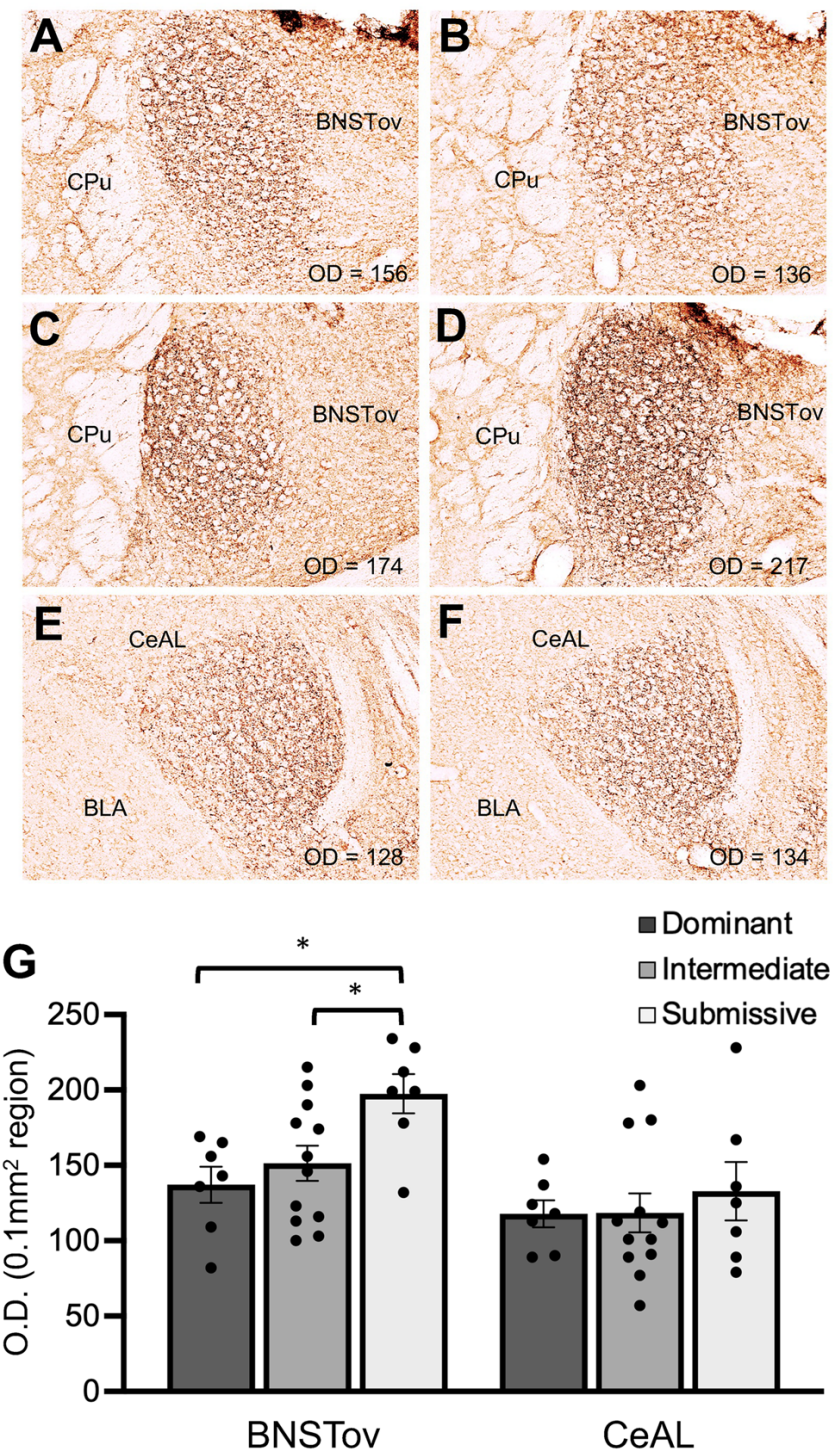
Representative coronal sections through the BNST (**A**–**D**) and CeA (**E**,**F**) showing PACAP IHC expression from Dominant (**A**,**E**), Intermediate (**B**,**C**) and Submissive (**D**,**F**) ranked mice. Number in lower right corner of each panel reflects the measured optical density (O.D.) value of PACAP expression in 0.1 mm^2^ regions in the BNSTov and CeAL from each section (see Supplemental Fig. 2 for anatomical location of within the BNSTov and CeA where O.D. was measured). (**G**) Average O.D. values of PACAP expression in the BNSTov and CeAL for each group. Mice in the Submissive group showed significantly higher levels PACAP expression in the BNSTov compared to the other two groups; PACAP expression was not significantly different in the CeAL between groups. Bar graph data are shown as mean ± s.e.m. *CPu* Caudate-putamen, *BLA* basolateral amygdala. Scale bars = 100 mm. *P < 0.05.

A total of seven cages of mice were scored for agonistic behaviors at 12 weeks of age to rank animals into Dominant, Intermediate and Submissive groups and process their brains for PACAP IHC in the extended amygdala. Figure 2 shows representative brain sections from animals of different social rank illustrating PACAP IHC in the BNSTov and CeAL subdivisions which were quantification by optical density measurements of 0.1 mm^2^ regions in each of these areas. A two-way ANOVA of PACAP expression across groups showed a main effect of brain area (*F*_1,23_ = 20.8, P < 0.0001), indicating generally higher levels of PACAP expression in the BNSTov versus the CeAL, and social dominance group (*F*_2,23_ = 3.53, P < 0.05). The brain area x social dominance group interaction was not significant. One-way ANOVA of PACAP expression in the BNSTov showed a significant overall effect (*F*_2,23_ = 5.27, P < 0.05); individual pairwise comparisons showed significantly higher levels of PACAP in the BNSTov in Submissive versus Dominant and Intermediate mice (P < 0.05 both comparisons).

**Figure 3:**
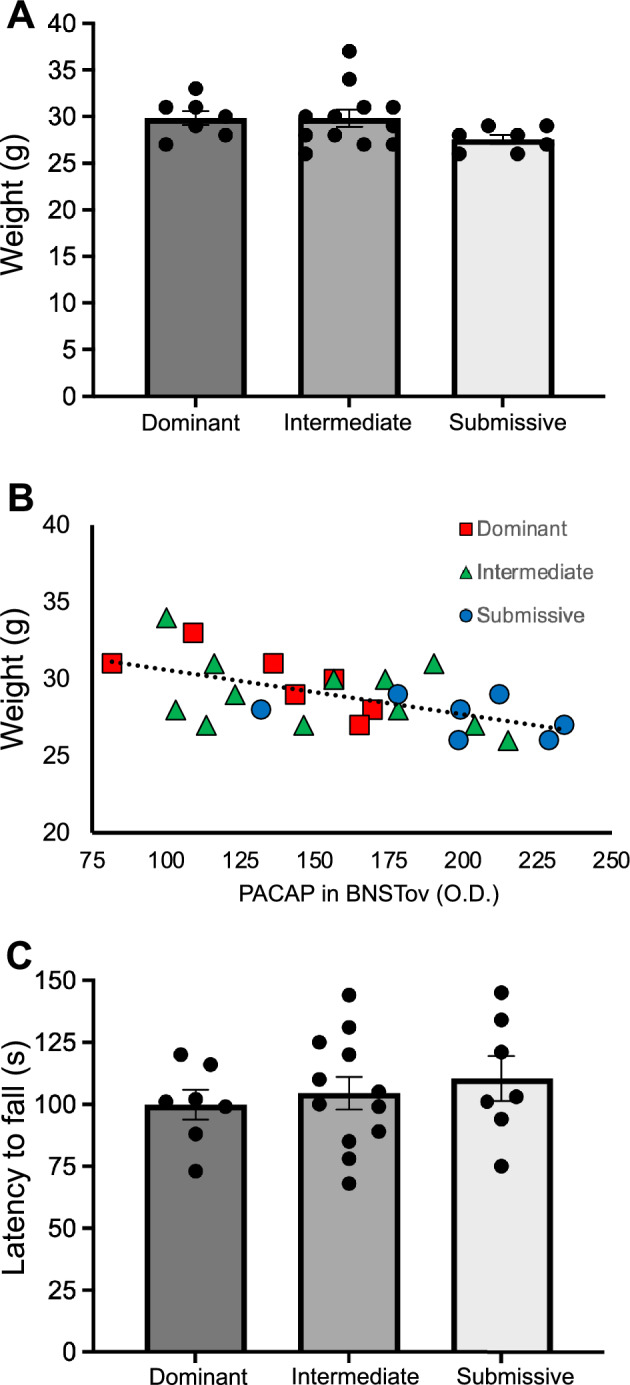
(**A**) Average body weight data from mice in the different social dominance groups at 12 weeks of age. (**B**) Relationship between body weight data and BNSTov PACAP expression (O.D.; optical density value from 0.1 mm^2^ region) for mice in the different social dominance groups; there was an overall trend for mice with lower body weight to have higher BNSTov PACAP expression levels (r^2^ = 0.34), but this correlation was not significant. (**C**) Average rotorod data from these same mice in the different social dominance groups. Bar graph data are shown as mean ± s.e.m.

Figure 3A illustrates average body weight data from animals in the different social dominance groups analyzed for PACAP IHC in the BNSTov and CeA shown in Fig. 2. A one-way ANOVA across groups revealed no significant main effect. Although there was a trend for Submissive animals to have lower average weight than Dominant or Intermediate animals, post-hoc comparisons with Sheffe’s test revealed no significant pairwise differences in body weight. We further analyzed these data to look for correlations between PACAP expression in the BNSTov—which was significantly higher in Submissive animals compared to the other two social dominance groups—and body weight; Fig. 3B illustrates this relationship. There was a trend for animals with lower body weight to have higher BNSTov PACAP expression levels (r^2^ = 0.34), but this correlation was not significant. Figure 3C illustrates rotorod data from these same animals in the different social dominance groups. A one-way ANOVA across groups revealed no significant main effect of latency to fall, the dependent measure of motor coordination in this test.

### Serum CORT levels after agonistic interactions

**Figure 4:**
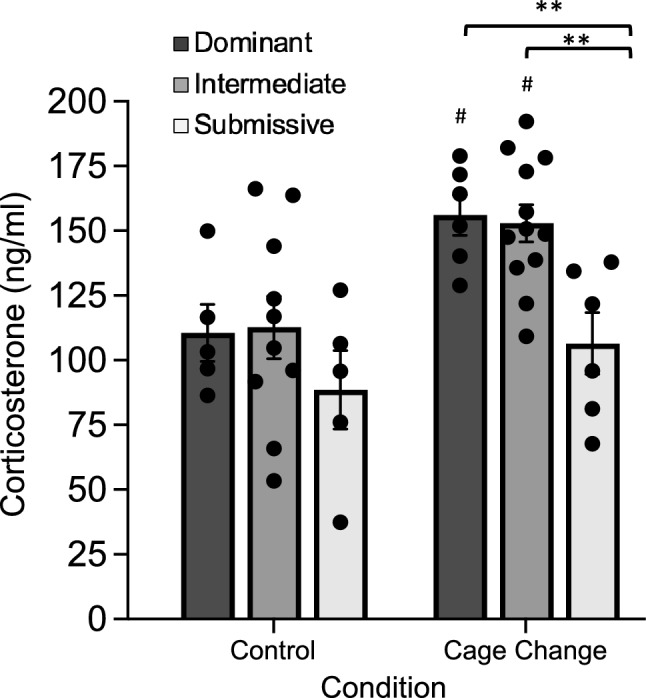
Serum corticosterone (CORT) levels from mice in the different social dominance groups measured under different cage change conditions. Groups of mice were sacrificed either immediately after the home cage change and 15 min recording session to identify Dominant, Intermediate and Submissive mice (Cage Change condition) or 48 h after this timepoint to measure CORT from undisturbed cages of mice (Control condition). Mice in the Dominant and Intermediate groups, but not Submissive group, showed significantly elevated CORT levels relative to controls (^#^P < 0.05) after cage change and social dominance interactions. After cage change, Submissive group mice showed significantly lower levels of CORT compared to other two groups (**P < 0.005 versus both groups) following this session. Bar graph data are shown as mean ± s.e.m.

A total of six cages of mice were used to examine blood serum CORT levels in Dominant, Intermediate and Submissive groups sacrificed immediately after mice engaged in agonistic interactions and behavior was recorded for identifying Dominant, Intermediate and Submissive mice following cage change at 12 weeks (Cage Change condition). An additional cohort of five cages of mice was used as controls to examine blood serum CORT levels in Dominant, Intermediate and Submissive groups identified at 12 weeks but were then undisturbed (i.e. no cage change) prior to sacrifice (48 h after 12 week cage change; Control condition). CORT data for each of the social dominance groups from both conditions are shown in Fig. 4. A between-subjects two-way ANOVA revealed a significant main effect of condition (*F*_1,38_ = 13.43, P < 0.005) and significant main effect of social dominance group (*F*_2,38_ = 6.33, P < 0.005). The condition x social dominance group interaction was not significant. Simple effects tests revealed that CORT levels were significantly higher after cage change in Dominant (*F*_1,38_ = 7.78, P < 0.05) and Intermediate (*F*_1,38_ = 6.1, P < 0.05) groups compared to respective control condition. CORT levels were not significantly different between the conditions for Submissive group mice indicating no effect of cage change/social interaction stress on CORT for this rank of mice. Separate one-way ANOVAs for each condition across social dominance groups revealed a significant main effect of CORT level in the cage change condition (*F*_2,21_ = 8.29, P < 0.005). Subsequent pairwise comparisons revealed significantly lower levels of CORT in Submissive mice compared to Dominant and Intermediate mice (P < 0.005 both comparisons).

### Social dominance and acoustic startle

**Figure 5:**
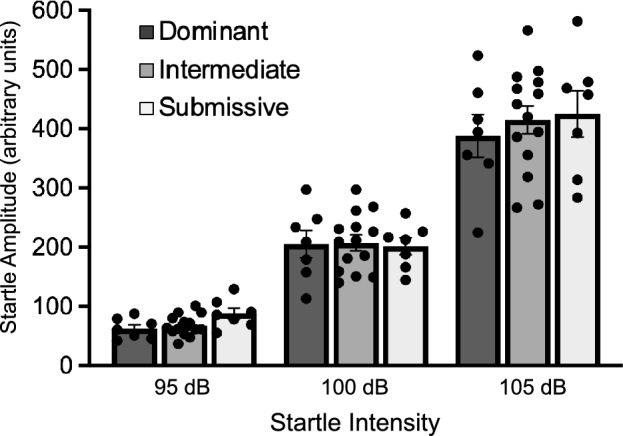
Acoustic startle response from mice in the different social dominance groups across startle intensity showing no differences between groups in this measure of sensorimotor reactivity. Bar graph data are shown as mean ± s.e.m.

Figure 5 shows acoustic startle data from 7 cages of mice ranked as Dominant, Intermediate and Submissive. A two-way ANOVA of startle amplitude for each of the startle eliciting intensities (95, 100 105 dB) across groups showed only a main effect of startle intensity (*F*_2,50_ = 309.2, P < 0.0001) as startle amplitude is increased with more intense startle stimuli.

## Discussion

The current study was designed to determine if there are relationships between expression of the stress-related neuropeptide PACAP in the extended amygdala and the social strata of group-housed C57BL/6 mice that occurs in the natural establishment of social dominance hierarchies among conspecific cagemates. Our hypothesis was that due to chronic, intermittent aggressive social interactions and naturalistic stress that animals experience over the nine weeks of group-housing, as mice self-organize into the social dominance hierarchies, there will be changes in stress-related factors that correspond with dominance rank. Social dominance rankings in the current study were based on the frequency of agonistic behaviors (e.g. aggressive and submissive behaviors) among group-housed mice following introduction into a new, unfamiliar homecage. Through these weekly perturbations in housing, mice re-establish and reveal stable dominance hierarchies that we presumed would have an underlying neurobiological signature in brain areas that subserve stress, threat detection, and adaptive responding, namely, the extended amygdala comprising the PACAP-dense regions of the BNST and CeA^48,59,60^. Accordingly, we found that expression of PACAP was highest in the BNSTov of the least-dominant (i.e., Submissive) mice compared to the most-dominant (i.e., Dominant) mice or mice ranked as neither most- or least-dominant (i.e., Intermediates). Dominant and Intermediate group mice showed similar levels of PACAP in the BNSTov. Interestingly, there were no differences in PACAP expression in the CeAL between any of the social dominance groups. Further, we examined if there was any impact of social dominance hierarchy on other dependent measures that are known to be influenced by stress and PACAP including body weight, CORT levels, and the acoustic startle response. Although Submissive mice tended to have lower body weight, there were no significant differences between groups. We found that cage change and accompanying bouts of agonistic behaviors stimulated CORT release in both Dominant- and Intermediate-ranked mice, but not Submissive mice, compared to an undisturbed control group where social dominance interactions were not initiated. Despite these changes in PACAP and CORT, we did not observe any differences between groups in sensorimotor reactivity of the acoustic startle response.

The finding of elevated PACAP expression in the BNST of Submissive group mice—cagemates that are the recipients of the greatest amount of aggressive behavior and respond with the greatest amount of defensive behavior—is consistent with previous reports showing that repeated bouts of stress increase both mRNA transcript and protein levels of PACAP in the BNST of rodents^36,61,62^. Unlike chronic stress, however, the effects of a single exposure to stress on PACAP levels in the BNST are mixed, as acute restraint stress has been shown to have no effect on PACAP mRNA levels^63^, whereas exposure to a series of footshocks significantly increases the density of PACAP peptide expression^52^. Differences in the quantitative and qualitative experience of the stress is likely to account for differential effects on adaptive regulation of PACAP in the BNST. In the current study, noxious visceral stimuli (e.g., bites, anogenital sniffing, pins) received by Submissive group mice would presumably be transmitted to the dorsal vagal complex (DVC) and lateral parabrachial nucleus (LPBN), where PACAP neurons reside and send projections to the BNST and CeA^46,64–67^. Hence, adaptations within this ascending circuit as a consequence of the repeated physical stress of aggressive engagements could account for the upregulation of PACAP peptide in the BNST seen in the Submissive group mice. While the BNST is traditionally known as a node for the integration of exteroceptive and interoceptive input related to stress that can regulate autonomic, neuroendocrine and behavioral phenotypes resembling anxiety states^68–70^, recent reports indicate that it also plays a role in coordinating social behavior^71^. We cannot determine from the current study as to the functional significance of increased PACAP expression in the BNST of Submissive group mice, but speculate that it may represent a neuroadaptation that provides some survival advantage to a low-ranking animal in the social dominance hierarchy. Along these lines, previous studies have shown that intra-BNST infusions PACAP elevates CORT levels in the blood^36^ and enhances acoustic startle reactivity^51,61^, which could be advantageous in helping animals increase arousal, attention and escape responding^72,73^. In contrast, other reports have shown that intra-BNST infusions of PACAP produce anorexia and loss of body weight which, at face value, would appear as maladaptive with potentially deleterious effects for the animal^74^. To better understand how our own findings of increased PACAP expression in the BNST in Submissive group mice fit in the context of these previous reports, we further examined the impact of homecage social dominance hierarchy on body weight, blood CORT levels, and acoustic startle (see below).

Our finding that PACAP expression levels in the CeA were not different between any of the social dominance groups, including Submissive group mice, aligns with a previous report showing that chronic stress (intermittent exposure to variable stressor) upregulates PACAP mRNA signal in the BNST, but not the CeA^61^. Further, others have shown that withdrawal following chronic intermittent ethanol dependence increases PACAP levels in the BNST but not the CeA^75^. Hence, there is an apparent dissociation between PACAP upregulation in the BNST versus the CeA which may be related to the qualitative nature of the type of stress experienced by animals. Despite that fact that both the BNST and CeA have been conceptualized as an interconnected neural continuum with similar afferent and efferent connections, including PACAPergic innervation, a dissociation between functional roles for these brain areas has been proposed^76^. According to this construct, the BNST plays a predominant role in sustained threat monitoring and may subserve behavioral phenotypes akin to anxiety (sustained fear) whereas the CeA is preferentially sensitive to phasic threats, mediating behaviors akin to acute fear^70,77^. In this context, and in relation to homecage social dominance hierarchies where subordinate animals may experience chronic intermittent stress as social rank is continually challenged and reinforced (i.e. sustained threat), we speculate that experience-dependent activation of BNST circuits, including PACAPergic pathways, may predominate over CeA circuits to transform the brains of animals of the lowest social dominance rank.

We also examined the impact of homecage social dominance rank on body weight given reports that, in general, group-housed subordinate animals have lower body weight than dominant animals^5,10,14,78^. While we did see a trend for Submissive group mice to have lower body weight than Dominant or Intermediate group mice, these differences were not significant. Further, we examined the relationship between BNSTov PACAP expression and body weight across all social dominance groups; there was a trend for animals with lower body weight to have higher PACAP expression in the BNSTov, but this correlation was not significant. Given that intra-BNST infusion of PACAP has been shown to induce anorexia and body weight loss^74^, we might have expected to see significantly lower body weight in Submissive group mice given our finding that these animals have increased BNSTov PACAP expression. However, a relatively high concentration of intra-BNST PACAP (1.0 μg) was required to induce significant weight loss in the prior report^74^ and it is unclear how comparable that is to the increase in endogenous PACAP in the BNSTov we observed in Submissive group mice such that it could have significantly impacted food intake and corresponding body weight in this group. Rotorod performance across all three dominance group was not significantly different indicating no impact of social dominance rank on motor coordination, as expected, based on previous reports assessing general motor performance in dominant versus subordinate animals^11,14,15^. We also examined the impact of social dominance hierarchy on the acoustic startle response, a sensorimotor reflex that is sensitive to repeated stress and anxiety-like states^31,51^ and is significantly increased by intra-BNST infusion of either CRF or PACAP^51,52,61,79^. However, we did not observe any differences in acoustic startle response between any of the social dominance groups.

Assessment of blood CORT is frequently used as an assay to explore the physiological effects of social dominance as it reflects activity of the descending hypothalamic–pituitary–adrenal (HPA) axis and has traditionally been thought of as a biomarker for stress and healthy functioning of this important feedback system^80^. Published data, however, are mixed as to the relationship between CORT levels and social dominance rank as some reports indicate subordinate animals have higher levels of CORT than dominant animals, whereas others report the opposite, or no change at all^54,78^. Differences between findings are likely related to the use of different strains, methods for dominance assessment, and stress-history of the animals^11^. In the current study we found that in undisturbed cages of mice, there were no differences in the resting level of CORT between the social dominance groups. However, following a cage change and the ensuing social dominance engagements, both the Dominant and Intermediate group show a significant elevation in CORT compared to the control condition, whereas Submissive group mice show similar CORT levels between the conditions. This suggests that Submissive group mice exhibit a blunted CORT response compared to the other two dominance groups in response to the stress of cage change and engagement in homecage social dominance behaviors. A related finding has been reported in rats showing that a subgroup of subordinate-ranked animals failed to mount the appropriate surge in CORT levels following exposure to a stressor^5^. This subgroup of “non-responders” made up approximately 40% of rats ranked as subordinate, suggesting that subordination can have profound effects on the adaptive function of the neuroendocrine system responsible for an organism’s ability to respond to stressful conditions^5^. In a more recent report using an assessment to rank group-housed male mice (4/cage) as dominant or subordinate similar to ours (e.g. scoring of homecage agonistic behaviors), the mice ranked lowest in the dominance hierarchy had lower serum CORT levels than dominant mice, although the effects of acute stressor on CORT was not assessed to determine if there is a blunted response in subordinate mice^14^.

Given our observation of increased PACAP expression in the BNSTov of Submissive group mice, it is tempting to propose that there may be a causal relationship between this neuroanatomical finding and the finding of reduced CORT levels in this same subgroup. While it is known that the BNST plays a role in descending control of the HPA axis response to stress^81^, the heaviest projections from the BNST to the paraventricular nucleus of the hypothalamus (PVN; the first relay in the descending HPA axis) appear to originate from other subdivisions of the BNST, including the anterior ventral (av) and dorsomedial (dm) BNST, with only a weak projection from the BNSTov^81–87^. However, there is evidence that CRF neurons in the BNSTov do project directly to the PVN in mice and rats^88^. This projection is likely relevant because PACAPergic afferents in the BNSTov make direct contact with CRF neurons in this area^89^ and presumably influence their activity. Further, because CRF neurons in the BNST are known to also co-express GABA^90^, it is possible that PACAP in the BNSTov modulates inhibitory tone in the PVN through this same projection^91^. Hence, enhanced PACAPergic innervation of the BNSTov, as seen in Submissive group mice, might affect PVN activity via enhanced CRF/GABA release and potentially serve to inhibit activity of the HPA axis to blunt CORT release in response to stress. Such a mechanism, however, would need to be reconciled with seemingly contradictory findings that (1) direct intra-BNST infusion of PACAP can significantly increase CORT levels^36^ and (2) elevated CORT in response to social defeat stress is attenuated in PACAP knockout mice^92^. In addition, it has been reported that another source of PACAP innervation of the BNST is from the PVN itself, suggesting the potential for reciprocal control of BNST-PVN feedback circuits under the control of PACAP^67^. Our current studies provide the basis for future mechanistic studies to characterize these circuits and their roles in social dominance.

Clearly, the multiple parallel and intersecting systems that underlie adaptive control of stress and may be recruited in the development of social dominance is more complex than can be addressed here. However, we believe our current findings contribute new insight into the study of social dominance and the influence of stress peptides, such as PACAP, on these networks^9,53,93^. Of interest to us is how these systems may become pathologically dysregulated as a consequence of sustained threat and chronic intermittent stress like that experienced by subordinates in the context of social dominance hierarchies. Bullying in children and teenagers is one such manifestation of social dominance that has demonstrable adverse effects on mental health in subordinated youth, with greater incidence of depression, anxiety, self-harm and suicidality in victims^94,95^. Hence, developing preclinical models with improved face and construct validity to study the impact of social dominance may lead to transformative advances in our understanding of human psychiatric conditions associated with social dominance and subordination, and enable studies in research animals to better predict outcomes in humans. While we did not uncover a behavioral phenotype (e.g. changes in acoustic startle) in the current study, we did find two significant effects that were idiosyncratic to submissive animals: (1) increased expression of PACAP peptide in the BNSTov and (2) normal resting levels of CORT but a blunted response following mild stress instigated by agonistic interactions. The later finding is interesting as blunted cortisol reactivity in response to stress has been associated with major depression, including in children^96,97^. Taken together, these observations made in low dominance-ranking animals may have translational value in an effort to elucidate pathologies associated with the adverse consequences of social dominance in humans, and potentially identify better treatments for disorders that arise from these experiences.

### Supplementary Information

Supplementary Figure 1.

Supplementary Figure 2.

Supplementary Legends.

## Data Availability

The datasets generated during and/or analyzed during the current study are available from the corresponding author on reasonable request.

## References

[1] Chou Y-J, Ma Y-K, Lu Y-H, King J-T, Tasi W-S, Yang S-B, Kuo T-H (2022). Potential cross-species correlations in social hierarchy and memory between mice and young children. Commun. Biol..

[2] Hu X, Wang T, Huang D, Wang Y, Li Q (2021). Impact of social class on health: The mediating role of health self-management. PLoS ONE.

[3] Sapolsky RM (2004). Social status and health in humans and other animals. Annu. Rev. Anthropol..

[4] Snyder-Mackler N, Burger JR, Gaydosh L, Belsky DW, Noppert GA, Campos FA, Bartolomucci A, Yang YC, Aiello AE, O’Rand A, Harris KM, Shively CA, Alberts SC, Tung J (2020). Social determinants of health and survival in humans and other animals. Science.

[5] Blanchard DC, Sakai RR, McEwen B, Weiss SM, Blanchard RJ (1993). Subordination stress: Behavioral, brain, and neuroendocrince correlates. Behav. Brain Res..

[6] Drews C (1993). The concept and definition of dominance in animal behavior. Behavior.

[7] Larrieu T, Cherix A, Duque A, Rodrigues J, Lei H, Gruetter R, Sandi C (2017). Hierarchical status predicts behavioral vulnerability and nucleus accumbens metabolic profile following chronic social defeat stress. Curr. Biol..

[8] Varholick JA, Bailoo JD, Palme R, Wurbel H (2018). Phenotypic variability between social dominance ranks in laboratory mice. Sci. Rep..

[9] Zhou T, Sandi C, Hu H (2018). Advances in understanding neural mechanisms of social dominance. Curr. Opin. Neurobiol..

[10] Fulenwider HD, Caruso MA, Ryabinin AE (2020). Manifestations of domination: Assessments of social dominance in rodents. Genes Brain Behav..

[11] Varholick JA, Bailoo JD, Jenkins A, Voelkl B, Wurbel H (2021). A systematic review and meta-analysis of the relationship between social dominance status and common behavioral phenotypes in male laboratory mice. Front. Behav. Neurosci..

[12] Wang F, Kessels HW, Hu H (2014). The mouse that roared: Neural mechanisms of social hierarchy. Trends Neurosci..

[13] Barabas AJ, Lucas JR, Erasmus MA, Cheng H-W, Gaskill BN (2021). Who’s the boss? Assessing convergent validity of aggression based dominance measures in male laboratory mice, *Mus musculus*. Front. Vet. Sci..

[14] Horii Y, Nagasawa T, Sakakibara H, Takahashi A, Tanave A, Matsumoto Y, Nagayama H, Yoshimi K, Yasuda MT, Shimoi K, Koide T (2017). Hierarchy in the home cage affects behaviour and gene expression in group-housed C57BL/6 male mice. Sci. Rep..

[15] Bartolomucci A, Palanza P, Gaspani L, Limiroli E, Panerai AE, Ceresini G, Poli MD, Parmigiani S (2001). Social status in mice: Behavioral, endocrine and immune changes are context dependent. Physiol. Behav..

[16] Karamihalev S, Brivio E, Flachskamm C, Stoffel R, Schmidt MV, Chen A (2020). Social dominance mediates behavioral adaptation to chronic stress in a sex-specific manner. eLife.

[17] Kunkel T, Wang H (2018). Socially dominant mice in C57BL6 background show increased social motivation. Behav. Brain Res..

[18] Dwortz MF, Curley JP, Tye KM, Padilla-Coreano N (2021). Neural systems that facilitate the representation of social rank. Philos. Trans. R. Soc. Lond. B.

[19] Shan Q, Hu Y, Chen S, Tian Y (2021). Nucleus accumbens dichotomically controls social dominance in male mice. Neuropsychopharmacology.

[20] Wang F, Zhu J, Zhu Q, Lin Z, Hu H (2011). Bidirectional control of social hierarchy by synaptic efficacy in medial prefrontal cortex. Science.

[21] Greenberg GD, Howerton CL, Trainor BC (2014). Fighting in the home cage: Agonistic encounters and effects on neurobiological markers within the social decision-making network of house mice (*Mus musculus*). Neurosci. Lett..

[22] Lee W, Hiura LC, Yang E, Broekman KA, Ophir AG, Curley JP (2019). Social status in mouse social hierarchies is associated with variation in oxytocin and vasopressin 1a receptor densities. Hormones Behav..

[23] Lee W, Milewski TM, Dwortz MF, Young RL, Gaudet AD, Fonken LK, Champagne FA, Curley JP (2022). Distinct immune and transcriptome profiles in dominant versus subordinate males in mouse social hierarchies. Brain Behav. Immunity.

[24] So N, Franks B, Lim S, Curley JP (2015). A social network approach reveals associations between mouse social dominance and brain gene expression. PLoS ONE.

[25] Meloni EG, Gerety LP, Knoll AT, Cohen BM, Carlezon WA (2006). Behavioral and anatomical interactions between dopamine and corticotropin-releasing factor in the rat. J. Neurosci..

[26] Meloni EG, Reedy CL, Cohen BM, Carlezon WA (2008). Activation of raphe efferents to the medial prefrontal cortex by corticotropin-releasing factor: Correlation with anxiety-like behavior. Biol. Psychiatry.

[27] Van’t Veer A, Carlezon WA (2013). Role of kappa-opioid receptors in stress and anxiety-related behavior. Psychopharmacology.

[28] Bale TL, Vale WW (2004). CRF and CRF receptors: Role in stress responsivity and other behaviors. Annu. Rev. Pharmacol. Toxicol..

[29] Chudoba R, Dabrowski J (2023). Distinct populations of corticotropin-releasing factor (CRF) neurons mediate divergent yet complementary defensive behaviors in response to a threat. Neuropharmacology.

[30] Hostetler CM, Ryabinin AE (2013). The CRF system and social behavior: A review. Front. Neurosci..

[31] Hammack SE, May V (2014). Pituitary adenylate cyclase activating polypeptide in stress-related disorders: Data convergence from animal and human studies. Biol. Psychiatry.

[32] Gray SL, Cline DL (2019). PACAP: Regulator of the stress response. Stress.

[33] Stroth N, Holighaus Y, Ait-Ali D, Eiden LE (2011). PACAP: A master regulator of neuroendocrine stress circuits and the cellular stress response. Ann. N. Y. Acad. Sci..

[34] Agarwal A, Halvorson LM, Legradi G (2005). Pituitary adenylate cyclase-activating polypeptide (PACAP) mimics neuroendocrine and behavioral manifestations of stress: Evidence for PKA-mediated expression of the corticotropin-releasing hormone (CRH) gene. Mol. Brain Res..

[35] Grinevich V, Fournier A, Pelletier G (1997). Effects of pituitary adenylate cyclase-activating polypeptide (PACAP) on corticotropin-releasing hormone (CRH) gene expression in the rat hypothalamic paraventricular nucleus. Brain Res..

[36] Lezak KR, Roelke E, Harris OM, Choi I, Edwards S, Gick N, Cocchiaro G, Missig G, Roman CW, Braas KM, Toufexis DJ, May V, Hammack SE (2014). Pituitary adenylate cyclase-activating polypeptide (PACAP) in the bed nucleus of the stria terminalis (BNST) increases corticosterone in male and female rats. Psychoneuroendocrinology.

[37] Vaudry D, Falluel-Morel A, Bourgault S, Basille M, Burel D, Wurtz O, Fournier A, Chow BK, Hashimoto H, Galas L, Vaudry H (2009). Pituitary adenylate cyclase- activating polypeptide and its receptors: 20 years after the discovery. Pharmacol. Rev..

[38] Montero M, Yon L, Kikuyama S, Dufour S, Vaudry H (2000). Molecular evolution of the growth hormone-releasing hormone/pituitary adenylate cyclase-activating polypeptide gene family. Functional implication in the regulation of growth hormone secretion. J. Mol. Endocrinol..

[39] Alheid GF, Heimer L (1988). New perspectives in basal forebrain organization of special relevance for neuropsychiatric disorders: The striatopallidal, amygdaloid, and corticopetal components of substantia innominata. Neuroscience.

[40] Hannibal J (2002). Pituitary adenylate cyclase-activating peptide in the rat central nervous system: An immunohistochemical and in situ hybridization study. J. Comp. Neurol..

[41] Piggins HD, Stamp JA, Burns J, Rusak B, Semba K (1996). Distribution of pituitary adenylate cyclase activating polypeptide (PACAP) immunoreactivity in the hypothalamus and extended amygdala of the rat. J. Comp. Neurol..

[42] Boucher MN, May V, Braas KM, Hammack SE (2021). PACAP orchestration of stress-related responses in neural circuits. Peptides.

[43] Ferrara NC, Gilmartin MR (2020). Pituitary adenylate cyclase-activating polypeptide (PACAP) in stress, pain, and learning. Handb. Behav. Neurosci..

[44] Lebow MA, Chen A (2016). Overshadowed by the amygdala: The bed nucleus of the stria terminalis emerges as key to psychiatric disorders. Mol. Psychiatry.

[45] Cho JH, Zushida K, Shumyatsky GP, Carlezon WA, Meloni EG, Bolshakov VY (2012). Pituitary adenylate cyclase-activating polypeptide induces postsynaptically expressed potentiation in the intra-amygdala circuit. J. Neurosci..

[46] Missig G, Roman CW, Vizzard MA, Braas KM, Hammack SE, May V (2014). Parabrachial nucleus (PBn) pituitary adenylate cyclase activating polypeptide (PACAP) signaling in the amygdala: Implication for the sensory and behavioral effects of pain. Neuropharmacology.

[47] Meloni EG, Venkataraman A, Donahue RJ, Carlezon WA (2016). Bi-directional effects of pituitary adenylate cyclase-activating polypeptide (PACAP) on fear-related behavior and c-Fos expression after fear conditioning in rats. Psychoneuroendocrinology.

[48] Meloni EG, Kaye KT, Venataraman A, Carlezon WA (2019). PACAP increases Arc/Arg 3.1 expression within the extended amygdala after fear conditioning in rats. Neurobiol. Learn. Mem..

[49] Foilb AR, Taylor-Yeremeeva EM, Fritsch E, Ravichandran C, Lezak KR, Missig G, McCullough KM, Carlezon WA (2024). Differential effects of the stress peptides PACAP and CRF on sleep architecture in mice. NPP-Digit. Psychiatry Neurosc..

[50] Donahue RJ, Venkataraman A, Carroll FI, Meloni EG, Carlezon WA (2015). Pituitary adenylate cyclase-activating polypeptide disrupts motivation, social interaction, and attention in male Sprague Dawly rats. Biol. Psychiatry.

[51] Davis M, Walker DL, Lee Y (1997). Roles of the amygdala and bed nucleus of the stria terminalis in fear and anxiety measured with the acoustic startle reflex. Possible relevance to PTSD. Ann. N. Y. Acad. Sci..

[52] Seiglie MP, Huang L, Cottone P, Sabino V (2019). Role of the PACAP system of the extended amygdala in the acoustic startle response in rats. Neuropharmacology.

[53] Ferreira-Fernandes E, Peca J (2022). The neural circuit architecture of social hierarchy in rodents and primates. Front. Cell. Neurosci..

[54] Williamson CM, Lee W, Romeo RD, Curley JP (2017). Social context-dependent relationships between mouse dominant rank and plasma hormone levels. Physiol. Behav..

[55] Gong S, Miao Y-L, Jiao G-Z, Sun M-J, Li H, Lin J, Luo M-J, Tan J-H (2015). Dynamics and correlation of serum cortisol and corticosterone under different physiology or stressful conditions in mice. PLoS ONE.

[56] Kim JY, Kang HH, Ahn JH, Chung JW (2008). Circadian changes in serum corticosterone levels affect hearing in mice exposed to noise. Neuroreport.

[57] Arnold M, Langhans W (2010). Effects of anesthesia and blood sampling techniques on plasma metabolites and corticosterone in the rat. Physiol. Behav..

[58] Wu X-Y, Hu Y-T, Guo L, Zhu Q-B, Yu E, Wu J-L, Shi L-G, Huang M-L, Bao A-M (2015). Effect of pentobarbital and isoflurane on acute stress response in rat. Physiol. Behav..

[59] Kash TL, Pleil KE, Marcinkiewcz CA, Lowery-Gionta EG, Crowley N, Mazzone C, Sugam J, Hardaway JA, McElligott ZA (2015). Neuropeptide regulation of signaling and behavior in the BNST. Mol. Cells.

[60] Waraczynski M (2016). Towards a systems-oriented approach to the role of the extended amygdala in adaptive responding. Neurosci. Biobehav. Rev..

[61] Hammack SE, Cheung J, Rhodes KM, Schutz KC, Falls WA, Braas KM, May V (2009). Chronic stress increases pituitary adenylate cyclase-activating peptide (PACAP) and brain-derived neurotrophic factor (BDNF) mRNA expression in the bed nucleus of the stria terminalis (BNST): Roles for PACAP in anxiety-like behavior. Psychoneuroendocrinology.

[62] Roman CW, Lezak KR, Hartsock MJ, Falls WA, Braas KM, Howard AB, Hammack SE, May V (2014). PAC1 receptor antagonism in the bed nucleus of the stria terminalis (BNST) attenuates the endocrine and behavioral consequences of chronic stress. Psychoneuroendocrinology.

[63] Lezak KR, Roman CW, Braas KM, Schutz KC, Falls WA, Schulkin J, May V, Hammack SE (2014). Regulation of bed nucleus of the stria terminalis PACAP expression by stress and corticosterone. J. Mol. Neurosci..

[64] Boucher MN, Aktar M, Braas KM, May V, Hammack SE (2022). Activation of lateral parabrachial nucleus (LPBn) PACAP-expressing projections to the bed nucleus of the stria terminalis (BNST) enhances anxiety-like behavior. J. Mol. Neurosci..

[65] Chiang MC, Bowens A, Schier LA, Tupone D, Uddin O, Heinricher MM (2019). Parabrachial complex: A hub for pain and aversion. J. Neurosci..

[66] Jiang SZ, Zhang H, Eiden LE (2023). PACAP controls endocrine and behavioral stress responses via separate brain circuits. Biol. Psychiatry Glob. Open Sci..

[67] Kozicz T, Vigh S, Arimura A (1998). The source of origin of PACAP- and VIP-immunoreactive fibers in the laterodorsal division of the bed nucleus of the stria terminalis in the rat. Brain Res..

[68] Avery SN, Clauss JA, Blackford JU (2016). The human BNST: Functional role in anxiety and addiction. Neuropsychopharmacology.

[69] Crestani CC, Alves FHF, Gomes FV, Resstel LBM, Correa FMA, Herman JP (2013). Mechanisms in the bed nucleus of the stria terminalis involved in control of autonomic and neuroendocrine functions: A review. Curr. Neuropharmacol..

[70] Walker DL, Miles LA, Davis M (2009). Selective participation of the bed nucleus of the stria terminalis and CRF in sustained anxiety-like versus phasic fear-like responses. Prog. Neuropsychopharmacol. Biol. Psychiatry.

[71] Flanigan ME, Kash TL (2020). Coordination of social behaviors by the bed nucleus of the stria terminalis. Eur. J. Neurosci..

[72] Finsterwald C, Alberini CM (2014). Stress and glucocorticoid receptor-dependent mechanisms in long-term memory: From adaptive responses to psychopathologies. Neurobiol. Learn. Mem..

[73] Joels M (2018). Corticosteroids and the brain. J. Endocrinol..

[74] Kocho-Schellenberg M, Lezak KR, Harris OM, Roelke E, Gick N, Choi I, Edwards S, Wasserman E, Toufexis DJ, Braas KM, May V, Hammack SE (2014). PACAP in the BNST produces anorexia and weight loss in male and female rats. Neuropsychopharmacology.

[75] Ferragud A, Velazquez-Sanchez C, Minnig MA, Sabino V, Cottone P (2020). Pituitary adenylate cyclase-activating polypeptide (PACAP) modulates dependence-induced alcohol drinking and anxiety-like behavior in male rats. Neuropsychopharmacology.

[76] Davis M, Shi C (1999). The extended amygdala: Are the central nucleus of the amygdala and bed nucleus of the stria terminalis differentially involved in fear versus anxiety. Ann. N. Y. Acad. Sci..

[77] Davis M, Walker DL, Miles L, Grillon C (2010). Phasic vs sustained fear in rats and humans: Role of the extended amygdala in fear vs anxiety. Neuropsychopharmacology.

[78] Tamashiro KLK, Nguyen MMN, Sakai RR (2005). Social stress: From rodents to primates. Front. Neuroendocrinol..

[79] Lee Y, Davis M (1997). Role of the hippocampus, the bed nucleus of the stria terminalis, and the amygdala in the excitatory effect of corticotropin-releasing hormone on the acoustic startle reflex. J. Neurosci..

[80] Kinlein SA, Karatsoreos IN (2020). The hypothalamic-pituitary-adrenal axis as a substrate for stress resilience: Interactions with the circadian clock. Front. Neuroendocrinol..

[81] Choi DC, Furay AR, Evanson NK, Ulrich-Lai YM, Nguyen MMN, Ostrander MM, Herman JP (2008). The role of the posterior medial bed nucleus of the stria terminalis in modulating hypothalamic-pituitary-adrenocortical axis responsiveness to acute and chronic stress. Psychoneuroendocrinology.

[82] Barbier M, Gonzales JA, Houdayer C, Burdakov D, Risold P-Y, Croizier S (2020). Projections from the dorsomedial division of the bed nucleus of the stria terminalis to hypothalamic nuclei in the mouse. J. Comp. Neurol..

[83] Champagne D, Beaulieu J, Drolet G (1998). CRFergic innervation of the paraventricular nucleus of the rat hypothalamus: A tract-tracing study. J. Neuroendocrinol..

[84] Dong H-W, Petrovich GD, Watts AG, Swanson LW (2001). Basic organization of projections from the oval and fusiform nuclei of the bed nucleus of the stria terminalis in adult rat brain. J. Comp. Neurol..

[85] Dong H-W, Swanson LW (2006). Projections from the bed nuclei of the stria terminalis, dorsomedial nucleus: Implications for cerebral hemisphere integration of neuroendocrine, autonomic, and drinking responses. J. Comp. Neurol..

[86] Gungor NZ, Pare D (2016). Functional heterogeneity in the bed nucleus of the stria terminalis. J. Neurosci..

[87] Maita I, Bazer A, Blackford JU, Samuels BA (2021). Functional anatomy of the bed nucleus of the stria terminalis-hypothalamus neural circuitry: Implications for valence surveillance, addiction, feeding, and social behaviors. Handb. Clin. Neurol..

[88] Dabrowska J, Martinon D, Moaddab M, Rainnie DG (2016). Targeting corticotropin-releasing factor projections from the oval nucleus of the bed nucleus of the stria terminalis using cell-type specific neuronal tracing studies in mouse and rat brain. J. Neuroendocrinol..

[89] Kozicz T, Vigh S, Arimura A (1997). Axon terminalis containing PACAP-and VIP-immunoreactivity form synapses with CRF-immunoreactive neurons in the dorsolateral division of the bed nucleus of the stria terminalis in the rat. Brain Res..

[90] Partridge JG, Forcelli PA, Luo R, Cashdan JM, Schulkin J, Valentino RJ, Vicini S (2016). Stress increases GABAergic neurotransmission in CRF neurons of the central amygdala and bed nucleus stria terminalis. Neuropharmacology.

[91] Cullinan WE, Ziegler DR, Herman JP (2008). Functional role of local GABAergic influences on the HPA axis. Brain Struct. Funct..

[92] Lehmann ML, Mustafa T, Eiden AM, Herkenham M, Eiden LE (2013). PACAP-deficient mice show attenuated corticosterone secretion and fail to develop depressive behavior during chronic social defeat stress. Psychoneuroendocrinology.

[93] Milewski TM, Lee W, Champagne FA, Curley JP (2022). Behavioural and physiological plasticity in social hierarchies. Philos. Trans. R. Lond. B. Biol. Sci..

[94] Luo X, Zheng R, Xiao P, Xie X, Liu Q, Zhu K, Wu X, Xiang Z, Song R (2022). Relationship between school bullying and mental health status of adolescent students in China: A nationwide cross-sectional study. Asian J. Psychiatry.

[95] Halpern J, Jutte D, Colby J, Boyce WT (2014). Social dominance, school bullying, and child health: What are our ethical obligations to the very young?. Pediatrics.

[96] O’Connor DB, Thayer JF, Vedhara K (2021). Stress and health: A review of psychobiological processes. Annu. Rev. Psychol..

[97] Suzuki H, Belden AC, Sptznagel E, Dietrich R, Luby JL (2013). Blunted stress cortisol reactivity and failure to acclimate to familiar stress in depressed and sub-syndromal children. Psychiatry Res..

[98] Allen Institute for Brain Science. *Allen Mouse Brain Atlas [adult mouse; Coronal Images 52, 53, 54, 65,67,69, 71,74]*. https://www.mouse.brain-map.org (2004).

[99] Allen Institute for Brain Science. *Allen Reference Atlas—Mouse Brain [Adult Mouse; Coronal Images 52, 53, 54, 65,67,69, 71,74]*. https://www.atlas.brain-map.org (2011).

[100] Paxinos G, Franklin KBJ (2019). Paxinos and Franklin’s The Mouse Brain in Stereotaxic Coordinates.

