# Supplementary material for: Association between social dominance hierarchy and PACAP expression in the extended amygdala, corticosterone, and behavior in C57BL/6 male mice
Source: Sci Rep. 2024 Apr 18;14:8919. doi: 10.1038/s41598-024-59459-9 (PMC11026503; doi:10.1038/s41598-024-59459-9)

A

**Offensive behavior**	**Directs following behavior at another mouse**: chase, fight (reciprocal), bite (flank, neck or belly), anogenital sniffing, aggressive grooming, elicit submissive stance, mounts/pins
**Defensive behavior**	**Receives or engaged in following behavior**: fleeing, anogenital sniffing, bites, nuzzling/excessive grooming, being mounted, submissive posture (sit in upright position, underside exposed).

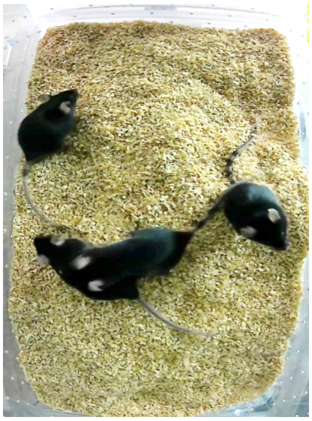

B

ID	O:Chase D:Flee	O: AG Sniff D: AG receive	O:Bite D: Bitten	O: Elicit submission/pin D: Submissive	O:Mount D:Mounted	O: Fight	Total O/D	Rank
M1	O:3 D:0	O:11 D:6	O:6 D:0	O:2 D:0	O:0 D:0	O:3	25/6	1 Dom
M2	O:1 D:0	O:8 D:4	O:2 D:0	O:0 D:0	O:0 D:0	O:3	14/4	2 Int1
M3	O:1 D:0	O:2 O:5	O:0 D:1	O:0 D:0	O:0 D:0	O:0	3/6	3 Int2
M4	O:0 D:5	O:4 D:10	O:0 D:7	O:0 D:2	O:0 D:0	O:0	4/24	4 Sub

C

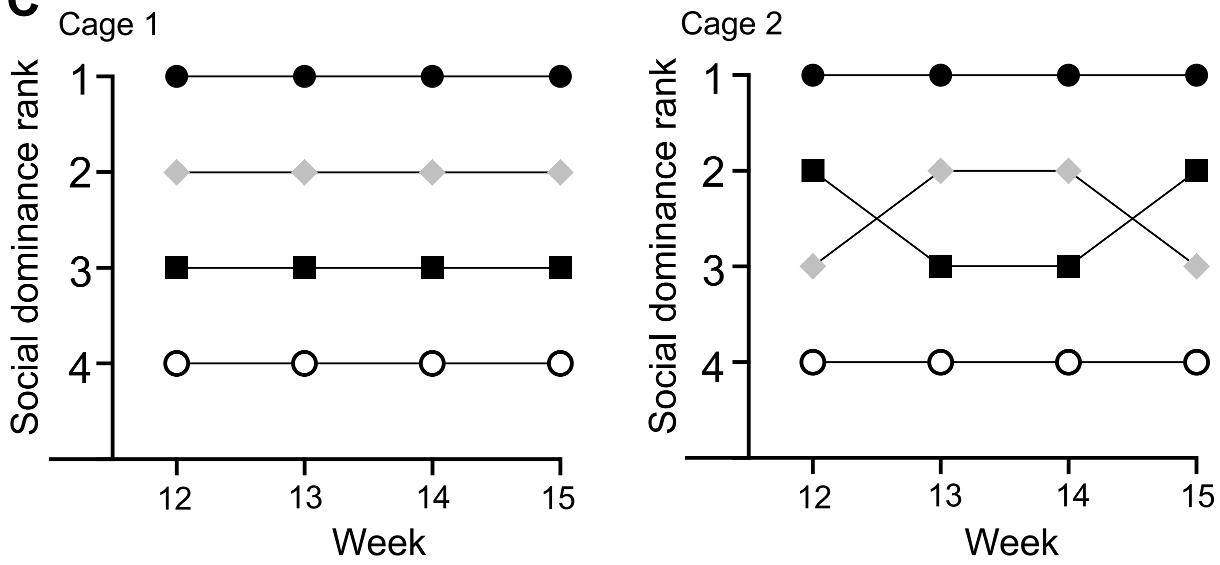

Supplement: Supplementary file 1 — Supplementary Figure 1. [file 41598_2024_59459_MOESM1_ESM.pdf]
