# Supplementary figures and images for: Association between social dominance hierarchy and PACAP expression in the extended amygdala, corticosterone, and behavior in C57BL/6 male mice
Source: Sci Rep. 2024 Apr 18;14:8919. doi: 10.1038/s41598-024-59459-9 (PMC11026503; doi:10.1038/s41598-024-59459-9)

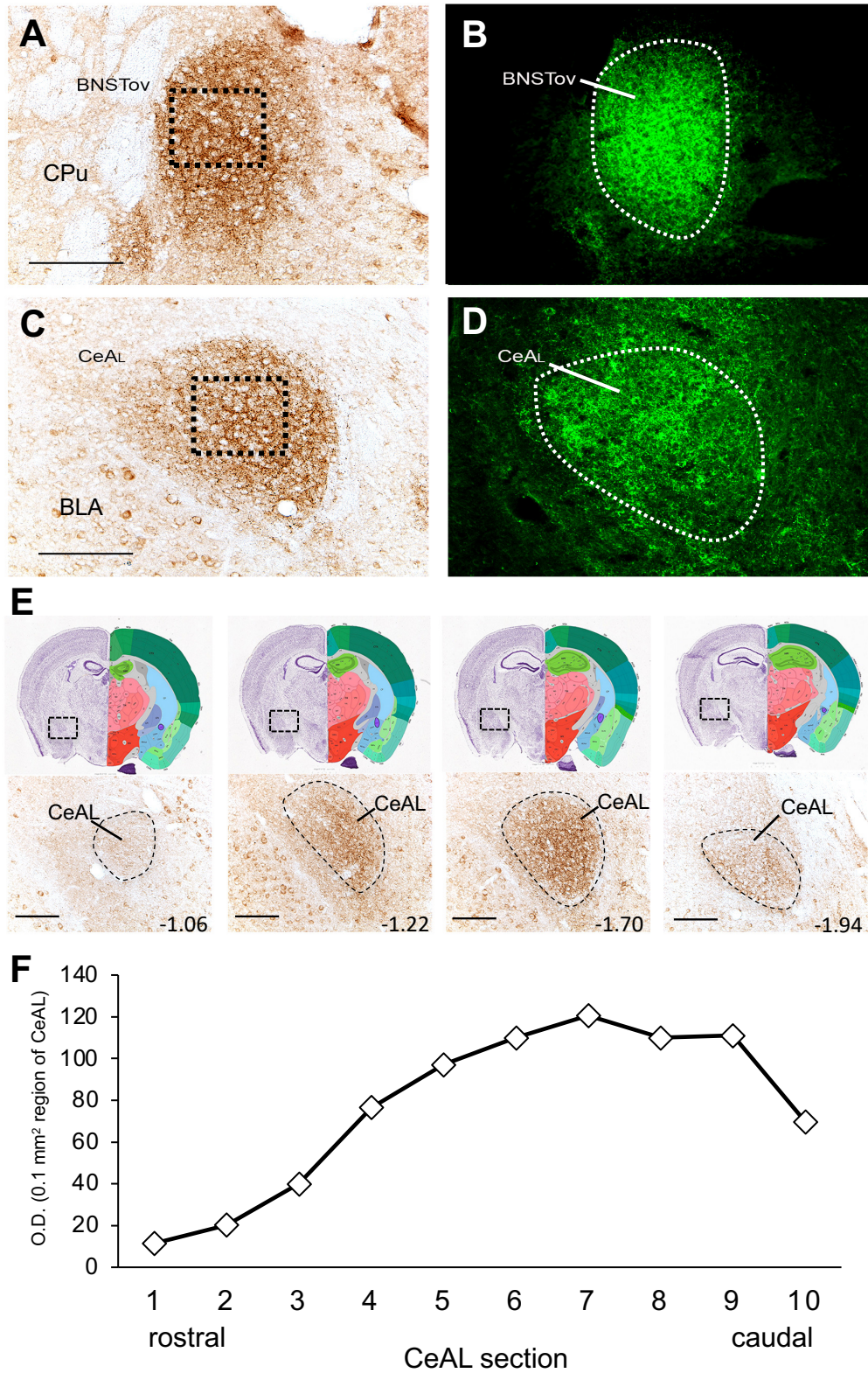

Supplement: Supplementary file 2 — Supplementary Figure 2. [file 41598_2024_59459_MOESM2_ESM.pdf]
