# Supplementary material for: Association between social dominance hierarchy and PACAP expression in the extended amygdala, corticosterone, and behavior in C57BL/6 male mice
Source: Sci Rep. 2024 Apr 18;14:8919. doi: 10.1038/s41598-024-59459-9 (PMC11026503; doi:10.1038/s41598-024-59459-9)
Supplement: Supplementary file 3 — Supplementary Legends. [file 41598_2024_59459_MOESM3_ESM.docx]

**Supplementary Figure 1. A.** List of offensive and defensive behaviors that were scored for each mouse (15 min observation) following cage change at 12 weeks and used to rank social dominance. *Right panel*: Example image of the homecage “arena” where behavior was recorded for scoring offensive and defensive behavior of each mouse housed 4/cage. **B.** Representative scorecard summarizing points given to each mouse (ID: M1, mouse 1; etc.) when observed engaging in offensive (O) or defensive (D) behaviors over the 15 min video recording session following cage change at 12 weeks. The overall score of offensive and defensive behaviors was used to rank animals as Dominant (Dom; highest offensive score), Intermediate (Int1,Int2) and Submissive (Sub; highest defensive score) mice. **C**. Data from two cages of mice (n=4/cage) where social dominance interactions were recorded weekly at weeks 12-15 following cage changes to assess the stability of the established social dominance hierarchy beyond the week 12 timepoint used to identify social dominance rank in this study . The longitudinal data indicate that social rank measured in this way is remarkably stable across time once established, especially for mice identified as either Dominant (rank 1) or Submissive (rank 4). Anogenital (AG).

**Supplementary Figure 2.** Representative coronal sections through the BNST (**A, B**) and CeA (**C, D**) showing PACAP expression (brown immunolabel); fluorescent images in **B** and **D** illustrate the approximate boundaries of the BNSTov and CeAL respectively. Optical density (O.D.) measurements of PACAP expression in 0.1 mm^2^ regions (boxes in A and C) in the BSNTov and CeAL were analyzed from brains of animals identified as Dominant, Intermediate or Submissive after scoring social dominance interactions at 12 weeks of age (see **Figure 2**).

**E.** Representative coronal sections through the rostro-caudal extent of the CeA showing differential expression levels of PACAP. *Top panel:* representative images from the Allen Mouse Brain Atlas showing the CeA at different rostro-caudal (left to right) levels; numbers in lower right corner of each image refer to the plate image from the Allen Brain Atlas [mouse.brain-map.org](http://mouse.brain-map.org/) and [atlas.brain-map.org](http://atlas.brain-map.org/). ^98,99^. Boxed area on left side (Nissl stained) of image represents magnified section in lower panels of showing PACAP expression (brown immunolabel) and approximate boundaries of CeAL at different rostro-caudal levels. Numbers in lower right corner of each image refer to the approximate brain level shown in millimeters posterior to bregma from the mouse brain stereotaxic atlas of Paxinos and Franklin (2019)^100^ for reference. **F.** Optical density (O.D.) measurements of 0.1 mm^2^ regions through the CeAL from serial (every third) sections through the CeA. As illustrated in the line graph, PACAP expression was strongest in caudal sections of the CeA. Scale bars = 100 μm.
